# Blood-cerebrospinal fluid barrier opening by modified single pulse transcranial focused shockwave

**DOI:** 10.1080/10717544.2022.2157068

**Published:** 2022-12-19

**Authors:** Yi Kung, Chueh-Hung Wu, Meng-Ting Lin, Wei-Hao Liao, Wen-Shiang Chen, Ming-Yen Hsiao

**Affiliations:** a Department of Biomechatronic Engineering, National Chiayi University, Chiayi City, Taiwan; b Department of Physical Medicine and Rehabilitation, National Taiwan University Hospital & National Taiwan University College of Medicine, Taipei City, Taiwan; c Institute of Biomedical Engineering and Nanomedicine, National Health Research Institutes, Miaoli, Taiwan

**Keywords:** Safety, shockwave, blood-cerebrospinal fluid barrier, CSF circulation, brain

## Abstract

Transcranial focused shockwave (FSW) is a novel noninvasive brain stimulation that can open blood-brain barriers (BBB) and blood-cerebrospinal fluid barriers (BCSFB) with a single low-energy (energy flux density 0.03 mJ/mm^2^) pulse and low-dose microbubbles (2 × 10^6^/kg). Similar to focused ultrasound, FSW deliver highly precise stimulation of discrete brain regions with adjustable focal lengths that essentially covers the whole brain. By opening the BCSFB, it allows for rapid widespread drug delivery to the whole brain by cerebrospinal fluid (CSF) circulation. Although no definite adverse effect or permeant injury was noted in our previous study, microscopic hemorrhage was infrequently observed. Safety concerns remain the major obstacle to further application of FSW in brain. To enhance its applicability, a modified single pulse FSW technique was established that present 100% opening rate but much less risk of adverse effect than previous methods. By moving the targeting area 2.5 mm more superficially on the left lateral ventricle as compared with the previous methods, the microscopic hemorrhage rate was reduced to zero. We systemically examine the safety profiles of the modified FSW-BCSFB opening regarding abnormal behavior and brain injury or hemorrhage 72 hr after 0, 1, and 10 pulses of FSW-treatment. Animal behavior, physiological monitor, and brain MRI were examined and recorded. Brain section histology was examined for hemorrhage, apoptosis, inflammation, oxidative stress related immunohistochemistry and biomarkers. The single pulse FSW group demonstrated no mortality or gross/microscopic hemorrhage (*N* = 30), and no observable changes in all examined outcomes, while 10 pulses of FSW was found to be associated with microscopic and temporary RBC extravasation (*N* = 6/30), and abnormal immunohistochemistry biomarkers which showed a trend of recovery at 72 hrs. The results suggest that single pulse low-energy FSW-BCSFB opening is effective, safe and poses minimal risk of injury to brain tissue (Sprague Dawley, SD rats).

## 1. Introduction

Blood–brain-barrier (BBB) opening by focused ultrasound (FUS) has been widely used in both animal models and clinical trials with prospective consequences for drug and gene delivery in central nervous system (CNS) related diseases (Lin et al., 2019; Meng et al., 2019). In addition to FUS, focused shockwave (FSW), a kind of acoustic wave with special modus, provides admirable dimensional resolution. Our previous studies develop an FSW-mediated BBB opening technique which achieves reversible and controllable BBB opening, then successfully transfect with a rat brain models (Kung et al., 2018, 2020). Nevertheless, both FUS and FSW-mediated BBB opening have limited effective drug delivery areas due to its millimeter scale of focal region, which may limit its applicability in various CNS diseases requiring widespread distribution of medication.

Aside from the BBB, the blood-cerebrospinal fluid barrier (BCSFB) at the boundary between the choroid plexuses and the ventricular system also prevents direct communication between the CNS and CSF. Compared with the BBB, the choroidal vessels in the BCSFB are extremely permeable. In addition the vessels are not surrounded by the pericytes and astrocytic foot processes (Yao et al., 2018). Once BCSFB is disrupted, drugs in the blood immediately enter the CSF encircling the entire CNS, thus potentially providing widespread and quick drug delivery (Montagne et al., 2020). It has been shown to effectively increase drug concentrations in the CSF to alleviate epilepsy (Kung et al., 2021, 2022). Furthermore, the FSW was administered to the lateral ventricle instead of brain parenchyma to induce BCSFB opening, offering lesser risk of adverse effect (Mestre et al., 2018). The method could potentially be used to treat CNS diseases which require widespread drug diffusion, such as meningitis, encephalitis, and neurogenerative diseases including Alzheimer’s disease.

Compared with FUS technique, the opening of BCSFB by single-pulse FSW requires only one-fifth of clinical dosage of microbubles (2 × 10^6^ MBs/kg, SonoVue) (Kung et al., 2021, 2022). In addition, FSW works at a lower frequency and thus offering better penetration with less attenuation (Kung et al., 2020). By using a single, short-duration FSW pulse, there is negligible thermal effect on target area.

Like high intensity pulse ultrasound (HIPU) and low intensity focused ultrasound (LIFU), FSW is well recognized for generating cavitation (Meng et al., 2019; Pasquinelli et al., 2019), which is believed to be the major mechanism responsible for barrier opening (Hsu et al., 2018). However, like FUS/FSW-BBB opening, therapeutic intensity ranges exist for the FSW-BCSFB opening technique above which tissue damage may occur due to inertial cavitation, and its safety has yet to be clarified (Blackmore et al., 2018). Although no definite adverse effect or permeant injury was noted in our previous study, safety concerns remain the major obstacle to further application of FSW in CNS. Particularly, microscopic hemorrhage was still observed, although infrequently, in our previous studies (Kung et al., 2021, 2022). Our preliminary studies indicated that more superficially targeted FSW was associated with less risk of hemorrhage. Thus, this study modified the single pulse FSW technique by adjusting the targeting area on the left lateral ventricle 2.5 mm more superficially as compared with the previous methods to further enhance its safety. We seek to systemically examine whether such modified FSW technique for the BCSFB opening causes behavioral or physiological abnormalities, or histology-proven CNS injury.

## 2. Materials and methods

### 2.1. Experimental animals

**Figure 1.**
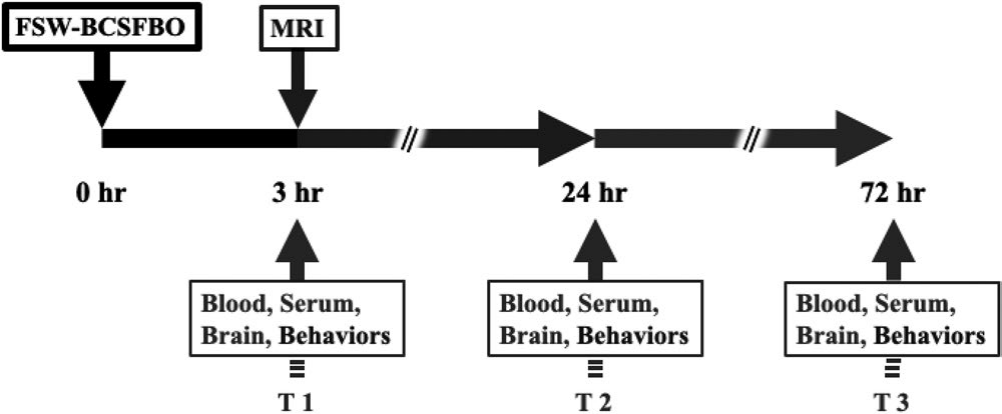
The experimental protocol. Safety evaluation was performed at 3 hrs (T 1), 24 hrs (T 2), and 72 hrs (T3) after applying FSW to induce BCSFB opening.

The rat used in this study was permitted by the ethics committee of the Laboratory Animal Center at the National Taiwan University College of Medicine (approval No. 20190361), and followed to the Animal Research: Reporting of In Vivo Experiments (ARRIVE) guidelines and to the Laboratory Animal Center Experimental Animal Care (LACEAC) guidelines. All rats (SD-rats between 8 and 10 weeks of age) were purchased from the LASCO Animal Center (Taiwan), fed with a standard diet and housed in a humidity (40–70%)-and temperature (19–23 °C)-controlled room with a 50% light-dark cycle. In order to evaluate the effect and safety of FSW-BCSFB opening, male SD-rats were assigned and randomized to 3 groups (control, single pulse, 10 pulses) according to the study design described below (Table 1, Figure 1). Severe hemorrhage was found after 10 pulses of FSW treatment in our previous studies (Kung et al., 2022). Therefore, 10 pulses of FSW were selected as a comparison.

**Table 1.** Summary of experimental animal parameters and analysis.

Group	Sub-group	FSW	Termination-time	Behavior-test	Terminated rats	Biomarkers analysis	Immunohistochemical analysis
		Pulses	Hrs	Number	Number	Number	Number
A	A1	0	3	30	10	5	5
	A2		24	20	10	5	5
	A3		72	10	10	5	5
B	B1	1	3	30	10	5	5
	B2		24	20	10	5	5
	B3		72	10	10	5	5
C	C1	10	3	30	10	5	5
	C2		24	20	10	5	5
	C3		72	10	10	5	5

### 2.2. FSW calibration

The acoustic pressure of the FSW device (PiezoWave, Richard Wolf, Germany) and its gel pad (F10 G4) employed in the current study was measured by a calibrated needle hydrophone (HGL-0400, ONDA, USA) in a free field within an acrylic tank filled with deionized and degassed water. The gel pad acts as a coupling agent as well as a spacer to determine the depth of FSW target. The measurement was done in 1 mm-steps, and the axial and cross sections field of view were 20 × 20 mm and 20 × 40 mm, respectively. The measured acoustic pressure of FSW corresponded to the intensity level of 0.1 of the manufacturer’s settings. The acoustic frequency of the FSW device ranges from 100 kHz to 20 MHz, with a peak frequency of 145 kHz analyzed by fast Fourier transform (WaveSurfer 3000/3000z Oscilloscopes, Lecroy).

### 2.3. FSW-induced BCSFB opening

**Figure 2.**
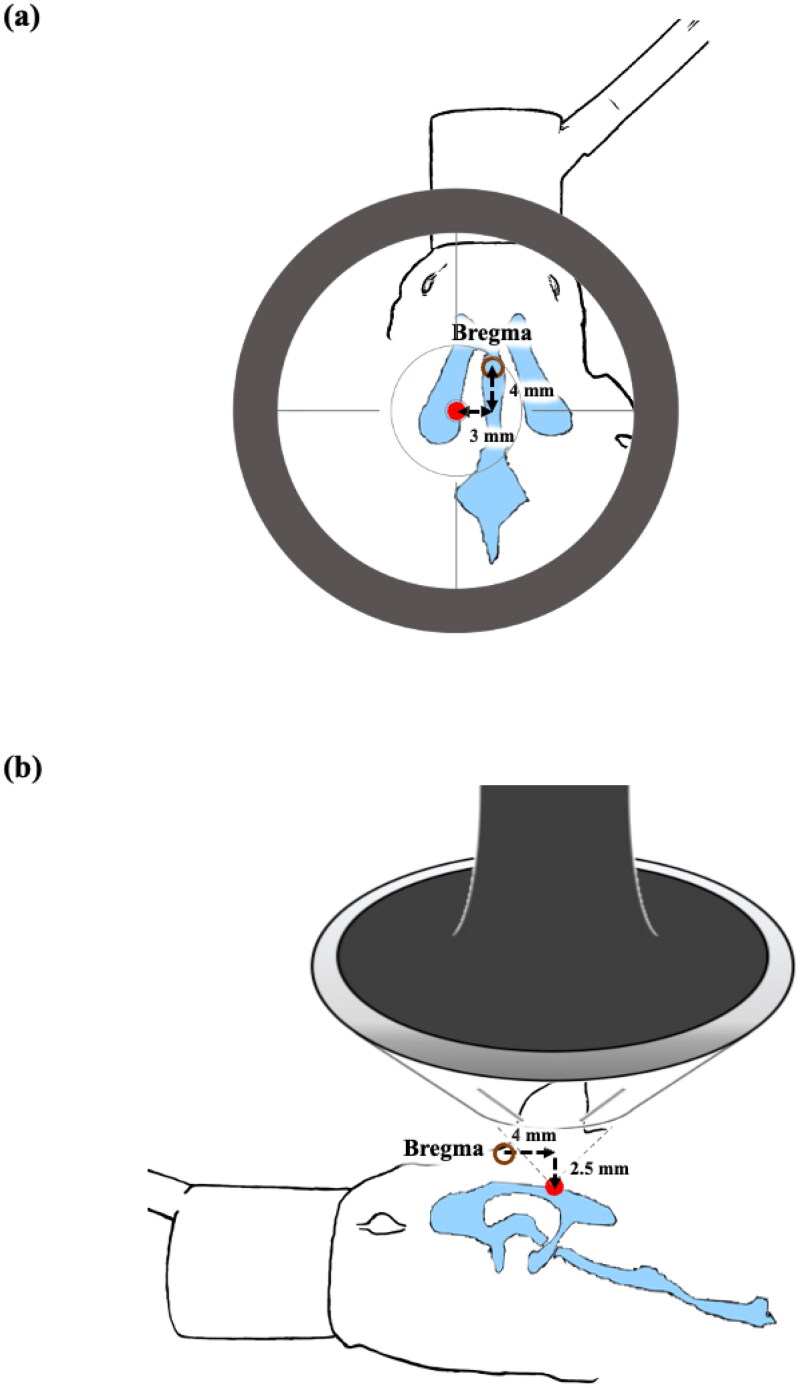
Positioning of the FSW probe, top view (a) and (b) lateral view. The focal point (red dot) is targeted at the choroid plexus on the lateral ventricles (AP 0, L 4, V − 5). The brown circle indicates the bregma.

As shown in Figure 2, the FSW probe was located on the top of the left lateral side of the rat head. The protocols of locating and setup of the FSW were altered from Kung et al. (2018, 2020). To lower the required acoustic energy of FSW stimulation to provide a safe operating range to open the BBB and BCSFB using the FSW-pulse technique, microbubbles (injected via the rats’ tail vein, 2 × 10^6^ MBs/kg, SonoVue, Diagnostics, Italy) are injected after implementing a single low-energy focused shockwave pulse (single pulse, intensity level 0.1, peak negative pressure −4.2 MPa; energy flux density 0.03 mJ/mm^2^).

The shockwave probe is 46 mm in radius, and the curvature radius is 62.9 mm. Composed with the gel pad, the shockwave probe ‘s focus is 5 mm from the bottom of the gel pad, targeting the left lateral ventricle (antero-posterior (AP) 0; mediolateral (ML) 4 mm; dorsoventral (DV) −2.5 mm). To reduce side effects, the targeting area was moved 2.5 mm more superficially on the left lateral ventricle as compared with the method described in previous studies (antero-posterior (AP) 0; mediolateral (ML) 4 mm; but the dorsoventral (DV) −5 mm (Paxinos & Watson, 1982; Kung et al., 2021).

For evaluating the Evans blue (EB) extravasation, 0.5 ml of 3% EB dye was injected via the rats’ tail vein after microbubbles injection. 3.5 hr after the FSW-BCSFB opening, the rats were sacrificed and the brains were harvested and sliced for analyzing the Evans blue extravasation.

For checking the recoverability, and estimating the duration of the FSW-BCSFB opening, 0.5 ml of 3% EB dye was infused intravenously 0.5, 1, 2, and 3 hr (*n* = 5) after FSW-BCSFB opening (the time zero point, 0 h). Then, samples of CSF were obtained 3.5 hr after time zero point to measure the concentration of EB dye.

### 2.4. Rat behavior tests and health monitors

Our previous results showed that a single FSW pulse consistently opens BCSFB with no detectable injury, on the other hand, 10 FSW pulses showed reversible erythrocyte extravasations. FSW openings of the BCSFB by a single pulse or by 10 pulses were performed at hour 0, and the rats were sacrificed at hours 3, 24, 72. Clinical parameters include: modified Irwin’s test plus body temperature, body weight, food consumption, hematology, and serum biochemistry Brain morphology and weights, and histology were also examined (Irwin, 1968; International Conference on Harmonisation, 2015).

Modified Irwin’s test was performed before and after FSW-treatment, including the motor activity, behavioral changes, coordination, sensory/motor reflex responses and body temperature of test subjects. The animal was observed and handled for 5 minutes the number of occurrences of descriptive and categorical endpoints (Table 2, and Table S1 and S2) were counted, scored and averaged for the 10 rats in each group. These experiments and rats were performed the Laboratory Animal Center of the National Taiwan University College of Medicine, a third-party research organization for preclinical testing, to ensure objectivity and fairness.

**Table 2.** Summarize percentage of opening rate and abnormal findings in control and FSW (×1 and ×10).

	Control	FSW (×1)	FSW (×10)
Successful BCSFBO rate	0.0 ± 0.0	100.0 ± 0.0^**,##^	100.0 ± 0.0^**,##^
Mortality	0.0 ± 0.0	0.0 ± 0.0	0.0 ± 0.0
Abnormal behavior	0.0 ± 0.0	0.0 ± 0.0	6.7 ± 25.4
Abnormal physiological monitor	0.0 ± 0.0	0.0 ± 0.0	0.0 ± 0.0
MRI (hyper intensity on T2WI)	0.0 ± 0.0	0.0 ± 0.0	0.0 ± 0.0^*,#^
Gross hemorrhage	0.0 ± 0.0	0.0 ± 0.0	0.0 ± 0.0
Microscopic hemorrhage	0.0 ± 0.0	0.0 ± 0.0	20.0 ± 42.2^*,#^
↑ Inflammation, oxidative stress and apoptosis-related biomarkers	0.0 ± 0.0	10.0 ± 30.5	30.0 ± 46.6
Abnormal serum biochemistry	0.0 ± 0.0	0.0 ± 0.0	0.0 ± 0.0

*^, #^and **^,# #^were *p* < 0.05 and *p* < 0.01vs. control and FSW (×1) in one-way ANOVA with the Tukey post hoc test.

Unit: %.

### 2.5. MRI and DWI analysis

MR images were obtained by 7-T MRI system (Biospec 70/30, Bruker, USA). Images were acquired with a field of view of 2.56 cm, a slice thickness of 1 mm, and a matrix size of 256 × 256. Images were zero-filled to 256 × 256, which results in an in-plane resolution of 100 μm × 100 μm. A fast spin-echo sequence was used (TR = 4000 ms, echo train length = 8, effective TE = 70 ms, NEX = 4) for T2-weighted imaging (T2WI) detection of brain edema. A warm-water blanket was used throughout the procedure to maintain the rat’s body temperature at 37 ◦C using, and the respiratory rate at 40–50 breaths per minute by altering the Forane levels. The region of interest (ROI) was set at the prefrontal cortex to quantify the signal intensity, t (Huang et al., 2021).

Diffusion-weighted imaging (DWI) was performed with three b-values of 20, 600 and 1200s/mm^2^ in three orthogonal directions along the *X*, *Y* and *Z* axes. Two 10 ms (δ) gradient pulses separated by 18 ms (Δ) on either side of the refocusing RF pulse were applied in a spin echo sequence with a 32 × 32 mm^2^ FOV, 128 × 128 matrix, 13 slices and 1 mm slice thickness. TR was 1500 ms and TE was 40 ms (Ding et al., 2010).

### 2.6. Histopathologic and immunohistochemical sections

Based on previous FUS-BBB opening studies, the term ‘adequate FSW exposure’ was defined as FSW application which produces consistent BCSFB opening without erythrocyte extravasation, or with limited extravasation, while the term ‘excessive FSW exposure’ was defined as FSW application that produces gross intracerebral hemorrhage (Tsai et al., 2018). To evaluate the effect of FSW-BCSFB opening on the rat brain tissue, after the rat behavior tests (section 2.4), the rats were sacrificed and then perfused with 4% paraformaldehyde for 24 hrs Subsequently, the tissue was embedded in paraffin for histology examinations.

H&E staining (ab245880, Abcam), GFAP detection (MAB360, Merck Millipore), TUNEL analysis (S7100, Merck Millipore), Caspase3-NeuN (AB3623 & MAB377, Merck Millipore) double staining were processed by the same protocols in our pervious study (Kung et al., 2021). For F4/80 staining, serial 4 µm paraffin sections were deparaffinized by EZ prep (Tucson, Ventana Medical Systems, Inc., USA). The slides were incubated with anti-F4/80 (polyclonal, Proteintech, China) in 1:2000 titration to form for 120 min using the automated Ventana Benchmark XT (Tucson, Ventana Medical Systems, Inc., USA). Labeling was detected with the Ultraview DAB Detection Kit (Tucson, Ventana Medical Systems, Inc., USA) as per the manufacturer’s protocol. All sections were counterstained with hematoxylin in Ventana reagent.

### 2.7. Biomarkers

The brains of the rats were removed 3, 24, 72 hrs after FSW induced BCSFB opening to evaluate the FSW dose effect between the FSW-BCSFB opening by a single pulse and by 10 pulses groups. Additionally, a blank group, 5 normal rat brains without FSW induced BCSFB opening were used to assess the difference of related biomarker expression in SOD (K335, BioVision), CAT (K773, BioVision), GSH (K261, BioVision), T-AOC (K274, BioVision), IL-1β (BSKR1006, Bioss Inc), MDA (E4601, BioVision) analysis (Kung et al., 2021). The dissected hippocampus was maintained at 4 °C. Subsequently, the homogenized brain tissues were analyzed as described as in the kit protocol.

### 2.8. Statistical analysis

Statistical analysis was achieved by the SPSS (v.26) statistical analysis. All data were present as mean ± standard deviation (SD) with at least 5 independent samples (*n*). One-way ANOVA was used for group comparisons, with Tukey post-hoc analysis). Less than 0.05 was considered as a significant *p*-value.

## 3. Results

### 3.1. Acoustic characterization of the FSW transducer

**Figure 3.**
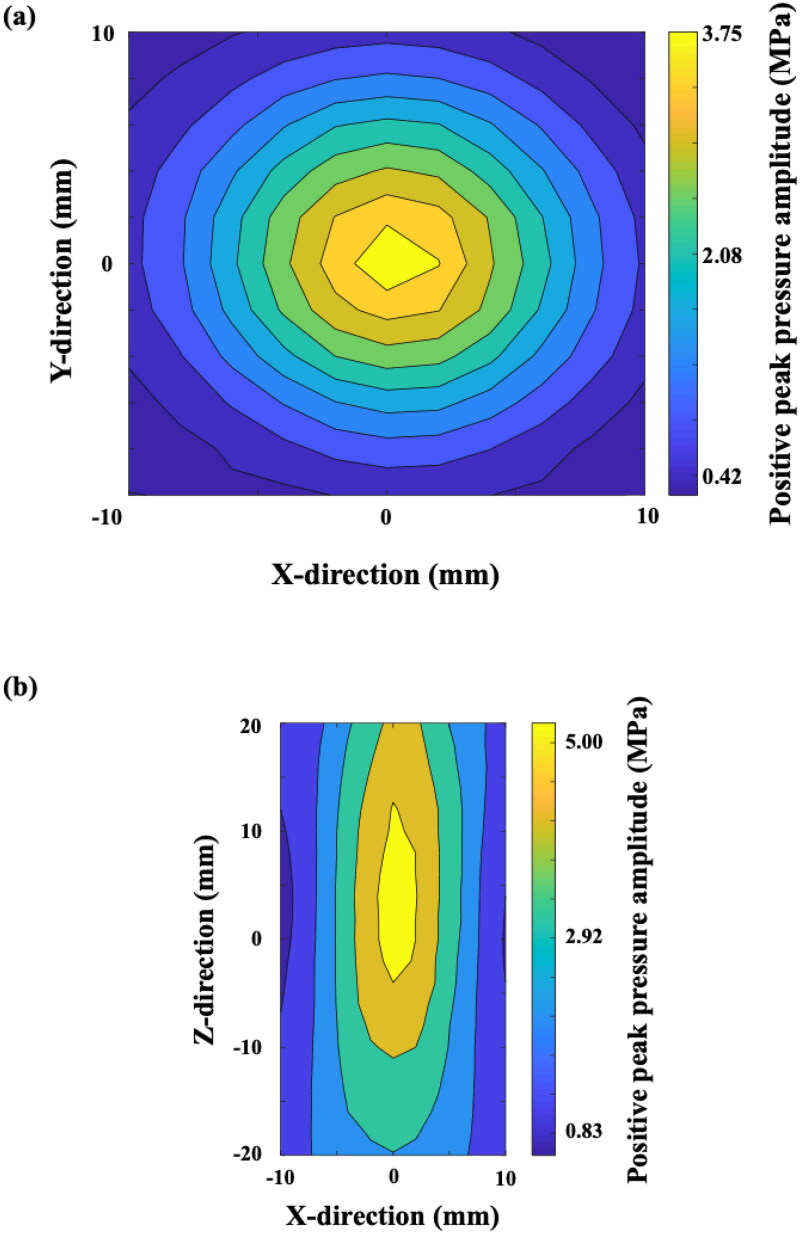
Positive peak pressure measurement of the FSW transducer. (a) free-field measurement on the X-Y directions; (b) free-field measurement on the X-Z directions. Intensity level: 0.1.

The FSW transducer (at intensity level: 0.1) without the gel-pad was characterized under linear propagation conditions with a calibrated hydrophone in degassed Q-water. Figure 3 showed the measured FSW focal pressure beam profiles along the X-Y and X-Z directions, indicating that the FSW focal distance was around 2.5 mm (from the surface of the transducer) with around 5.5 MPa of max positive peak pressure, and the lateral and axial full width half maximum (FWHM) pressure dimensions were around 5 mm and 18 mm, respectively.

### 3.2. BCSFB opening with FSW

**Figure 4.**
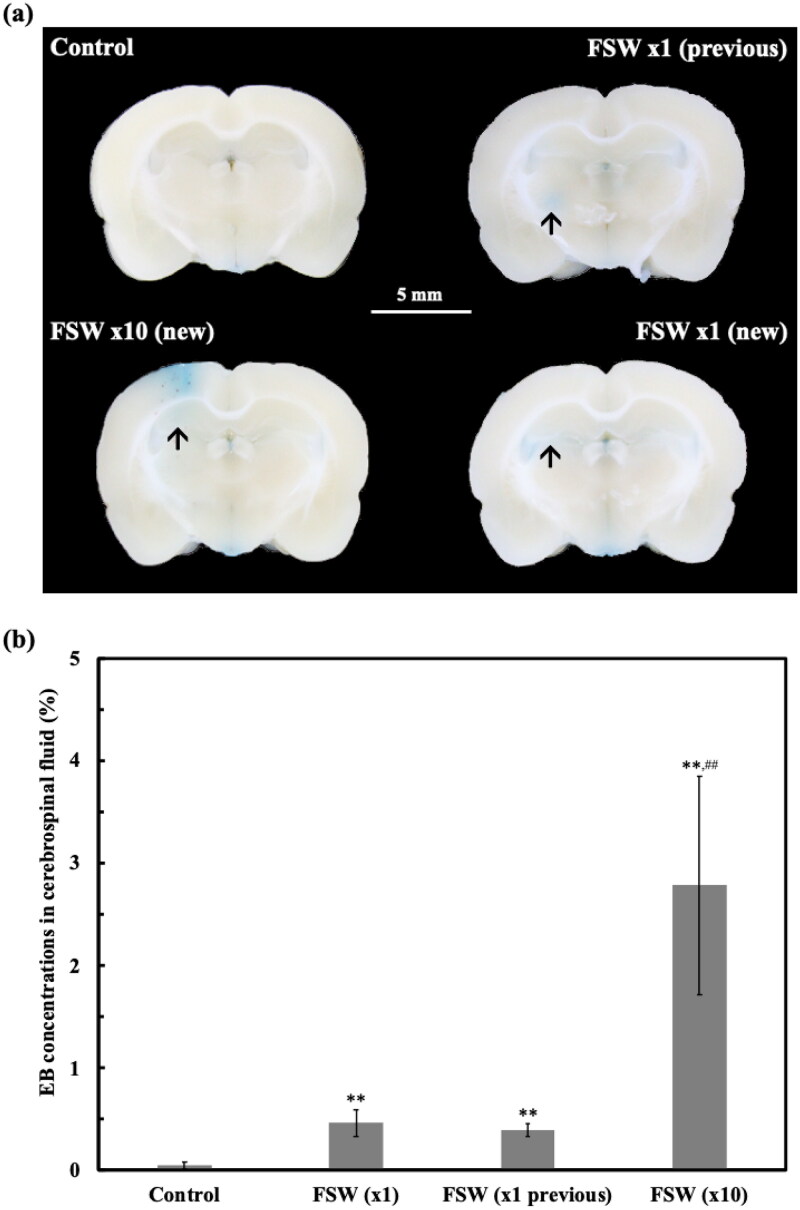
Evans blue persistence in the CSF after FSW-BCSFB opening, and control (no FSW). The current method applied the shockwave at the left lateral ventricle at 2.5 mm depth (antero-posterior (AP) 0; mediolateral (ML) 4 mm; dorsoventral (DV) –2.5 mm) while previous studies applied the shockwave at 5 mm depth (antero-posterior (AP) 0; mediolateral (ML) 4 mm; dorsoventral (DV) –5 mm). Other experimental conditions were the same between the current and previous studies. (a) Representative brain slices indicating positive BCSFB opening in both methods. The blue-stained areas were the Evans blue extravasation, indicating the BCSFB opening region (arrows); (b) The Evans blue concentration in the CSF increased significantly after FSW treatment in both methods. The CSF sampling time is at 3.5 hr after the FSW-treatment. **p, ##*p* < 0.01 vs. control and FSW-BCSFB opening (at same position), respectively. In one-way ANOVA with the Tukey post hoc test. (*n* = 5).

The single FSW achieve a 100% BCSFB opening rate (*N* = 30) as demonstrated by EB extravasation in brain section image (Figure 4(a)). The concentration ratio of Evans blue in CSF over blood was significantly elevated to 0.46 ± 0.13% in the FSW (×1) group and 2.78 ± 1.07% in the FSW (×10) group, as compared with control group (0.04 ± 0.04%) (Figure 4(b)).

Regarding the recoverability and the duration of the FSW-BCSFB opening, the EB concentrations in CSF was significantly elevated at 1 hr post-FSW-BCSFB opening (0.17 ± 0.06%), while there was no statistical significance between the 2 hr (0.07 ± 0.06%) group and control (0.04 ± 0.04%) (*n* = 5). This result indicates the FSW-BCSFB opening is recoverable with an opening duration of approximately 1 to 2 hr.

### 3.3. Adverse effect after FSW-BCSFB opening

The summarized percentage of abnormal findings in control and FSW (×1 and ×10) groups were shown on Table 2. Both FSW (×1) and FSW (×10) groups showed no abnormal brain MRI signals, gross hemorrhage, abnormal physiological monitoring or mortality. FSW (×1) group showed increased inflammation, oxidative stress and apoptosis-related biomarkers in 10% of brain sections at 24 hrs, that resolved at 72 hrs.

Microscopic hemorrhage occurred in none of FSW (×1) and in 20% of FSW (×10) group. The FSW (×10) group showed elevated inflammation, oxidative stress and apoptosis-related biomarkers were noted up to 72 hrs when compared to the control and FSW (×1) groups.

It’s worth noting that the modified FSW-BCSFB opening technique, by adjusting the FSW-targeted point from −5.0 mm to −2.5 mm (DV), achieved the same (100%) opening rate while resulted in much less adverse effect when compared with previous different FSW-barrier openings techniques, as summarized in Table 3. Particularly, there was no mortality gross hemorrhage or microscopic hemorrhage in the present method (DV= −2.5 mm, FSW (×1), *N* = 30), while microscopic hemorrhage was noted in 6.7% (*N* = 3/45) of DV= −5.0 mm, FSW (×1) group, and in 60.0% (*N* = 3/5) of DV= −5.0 mm, FSW (×10) group.

**Table 3.** Summary of FSW-BBB and BCSFB opening.

FSW								UCA		Focol-position				Barriers-opening			Adverse effect		
Intensity level	Pressure^+^ (MPa)	Pressure^−^ (MPa)	PRF (Hz)	Duration (Sec)	Pulse (no.)	Total energy (mJ)		Dose (Mega-MBs/kg)		AP (mm)	L (mm)	V (mm)		BBBO	BCSFBO		Mortality	Gross hemorrhage	Microscopic hemorrhage
0.1	5.4	−4.2	1	1	1	0.43		2		−4	3	−2.5		30/30	30/30		0 / 30	0 / 30	0 / 30
				10	10	4.3								30/30	30/30		0 / 30	0 / 30	6 / 30
0.1	5.4	−4.2	1	2	2	0.86		2		−4	3, −3	−5		5 / 5	5 / 5		0 / 5	0 / 5	1 / 5
				3	3	1.29				4, 0, −4	3	−5		5 / 5	5 / 5		1 / 5	1 / 5	2 / 5
				10	10	4.3				−4	3	−5		5 / 5	5 / 5		1 / 5	1 / 5	3 / 5
0.1	5.4	−4.2	1	1	1	0.43		2		4	3	−5		10 / 10	10 / 10		0 / 10	0 / 10	1 / 10
										0	3	−5		10 / 10	10 / 10		0 / 10	0 / 10	0 / 10
										−4	3	−5		45 / 45	45 / 45		0 / 45	0 / 45	3 / 45
										−4	6	−5		10 / 10	10 / 10		0 / 10	0 / 10	1 / 10
										−12	0	−5		10 / 10	10 / 10		6 / 10	8 / 10	10 / 10
										−16	0	−5		10 / 10	10 / 10		7 / 10	8 / 10	10 / 10
										0	3	−2.5		10 / 10	0 / 10		0 / 10	0 / 10	1 / 10
0.1	5.4	−4.2	1	1	1	0.43		0.1		0	3	−2.5		1 / 5	UK / 5		0 / 5	0 / 5	1 / 5
								1						2 / 5	UK / 5		0 / 5	0 / 5	2 / 5
								2						5 / 5	UK / 5		0 / 5	0 / 5	4 / 5
								5						5 / 5	UK / 5		0 / 5	0 / 5	5 / 5
1	11.80	−7.30	5	20	100	124.0		–		0	3	−2.5		0 / 5	UK / 5		0 / 5	0 / 5	0 / 5
3	15.51	−8.54				196.0								0 / 5	UK / 5		0 / 5	0 / 5	0 / 5
4	17.34	−9.17				233.0								1 / 5	UK / 5		0 / 5	0 / 5	1 / 5
5	19.22	−9.79	5	10	50	134.5		–		0	3	−2.5		2 / 8	UK / 8		0 / 8	0 / 8	1 / 8
				20	100	269.0								4 / 8	UK / 8		0 / 8	0 / 8	3 / 8
				40	200	538.0								5 / 8	UK / 8		0 / 8	0 / 8	5 / 8
				60	300	807.0								6 / 8	UK / 8		0 / 8	0 / 8	5 / 8
				80	400	1076.0								5 / 8	UK / 8		0 / 8	0 / 8	5 / 8
				100	500	1345.0								6 / 8	UK / 8		0 / 8	0 / 8	6 / 8
5	19.22	−9.79	5	10	50	134.5		0.0025		0	3	−2.5		0 / 5	UK / 5		0 / 5	0 / 5	0 / 5
								0.25						5 / 5	UK / 5		2 / 5	3 / 5	4 / 5
								2.5						5 / 5	UK / 5		2 / 5	3 / 5	5 / 5
								250						5 / 5	UK / 5		5 / 5	5 / 5	5 / 5
15	53.10	−15.80	1	1	1	6.96		–		0	3	−2.5		0 / 5	UK / 5		0 / 5	0 / 5	5 / 5
17	62.94	−16.96	1	1	1	7.85								1 / 5	UK / 5		0 / 5	1 / 5	5 / 5
20	77.70	−18.70	1	1	1	9.29								3 / 5	UK / 5		0 / 5	2 / 5	5 / 5

UK: Unknown.

### 3.4. Rat behavior tests and physiological monitors

After FSW-BCSFB opening procedure, the modified Irwin test was assessed. It has been shown that a single FSW pulse resulted in consistent BCSFB opening without noticeable erythrocyte extravasation, meanwhile, a sequence of 10 FSW pulses poses a risk for larger scale erythrocyte extravasations at 3 hr and 24 hr post-treatment. Therefore, we examined the behavior of SD rats subjected to FSW-BCSFB opening with a single pulse (minimal exposure), 10 pulses (excessive exposure), and untreated (control).

No animal mortality, hypoactivity, ataxia, tremors, or moribundity occurred during the study period (Table S1a). Additionally, no abnormal posture, stereotype, or behaviors were monitored during the study period (Table S1b). Touch- and click responses were observed on 2 rats out of the 30 subjected to 10 pulses of FSW-BCSFB opening at 3 hr after the treatment (Table S1c). Of note, these responses after FSW treatment were temporary that spontaneously resolved in the following observation. The behavior tests showed no significant between-group differences in the FSW and blank groups, indicating no neuromuscular dysfunction.

On the other hand, the physiological monitors including food consumption, body- and brain-weight, body temperature (Table S2a), serum biochemistry (Table S2b), and hematology (Table S2c) showed no significant changes, and all of those values were located on normal ranges of healthy rats (checked by a clinical veterinarian from a third-party research organization for preclinical testing).

### 3.5. MRI and DWI analysis

**Figure 5.**
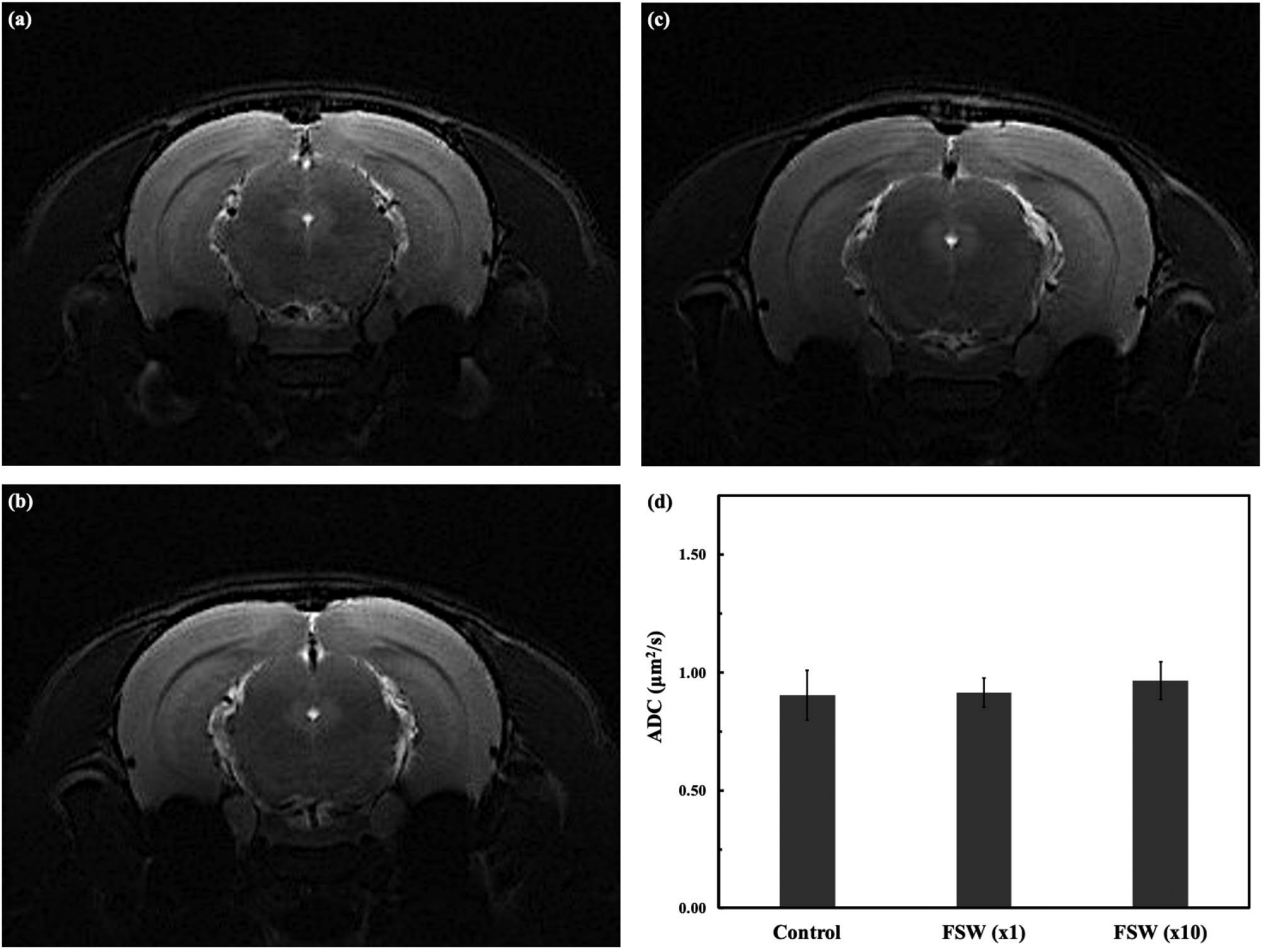
*In vivo* detection of brain edema formation at 3 hr after FSW treatment. (a) control, (b) FSW (×1), and (c) FSW (×10) groups; (d) comparisons of apparent diffusion coefficient (ADC) values of the control, FSW (×1), and FSW (×10) groups (*n* = 6).

T2W images (Figure 5a–c) and DWI analysis (Figure 5d) were acquired at 3 hr after implementing FSW pulse(s) on the left lateral ventricle. In this study, all the rats with/out FSW-treatment showed no hyper intensity on brain T2WI, which were used to indicate the lesions. Even though the ADC values on the DWI analysis of the FSW (×10) group were higher than other groups, the difference was not statistically significant.

### 3.6. Histopathologic and immunohistochemical sections

The histopathologic and immunohistochemical sections at the left lateral ventricle area showed differences in terms of the configuration and cell death at 3 hr and 24 hr after FSW (×10) treatments (Figure 6a–e). However, the risky conditions recovered at 72 hr. Quantitatively, the Caspase3-NeuN stains showed no significant difference in the control and the FSW (×1) groups during the entire observation period (Figure 6f). The FSW (×10) groups (Figure 6f) also showed that the recovery trend at 72 hr is the same as other histopathologic and immunohistochemical sections (Figure 6a–e). The erythrocyte extravasation ratio was 100% for both FSW (×1; ×10) groups. The microscopic hemorrhage ratios were 0.0% and 20.0% respectively, for FSW (×1; ×10) groups.

Figure 6.Comparisons of histopathologic and immunohistochemistrical sections by control, FSW (×1), and FSW (×10) groups. (a) H&E stains; (b) TUNEL stains; (c) GFAP stains;(d) F4/80 stains;(e) Caspase3-NeuN stains. Arrow, the cell stained positive for Caspase-3; (f) Caspase-3 density at different time points after FSW. *p < 0.05 vs. control groups in one-way ANOVA with the Tukey post hoc test. (*n* = 5).

**Figure:**
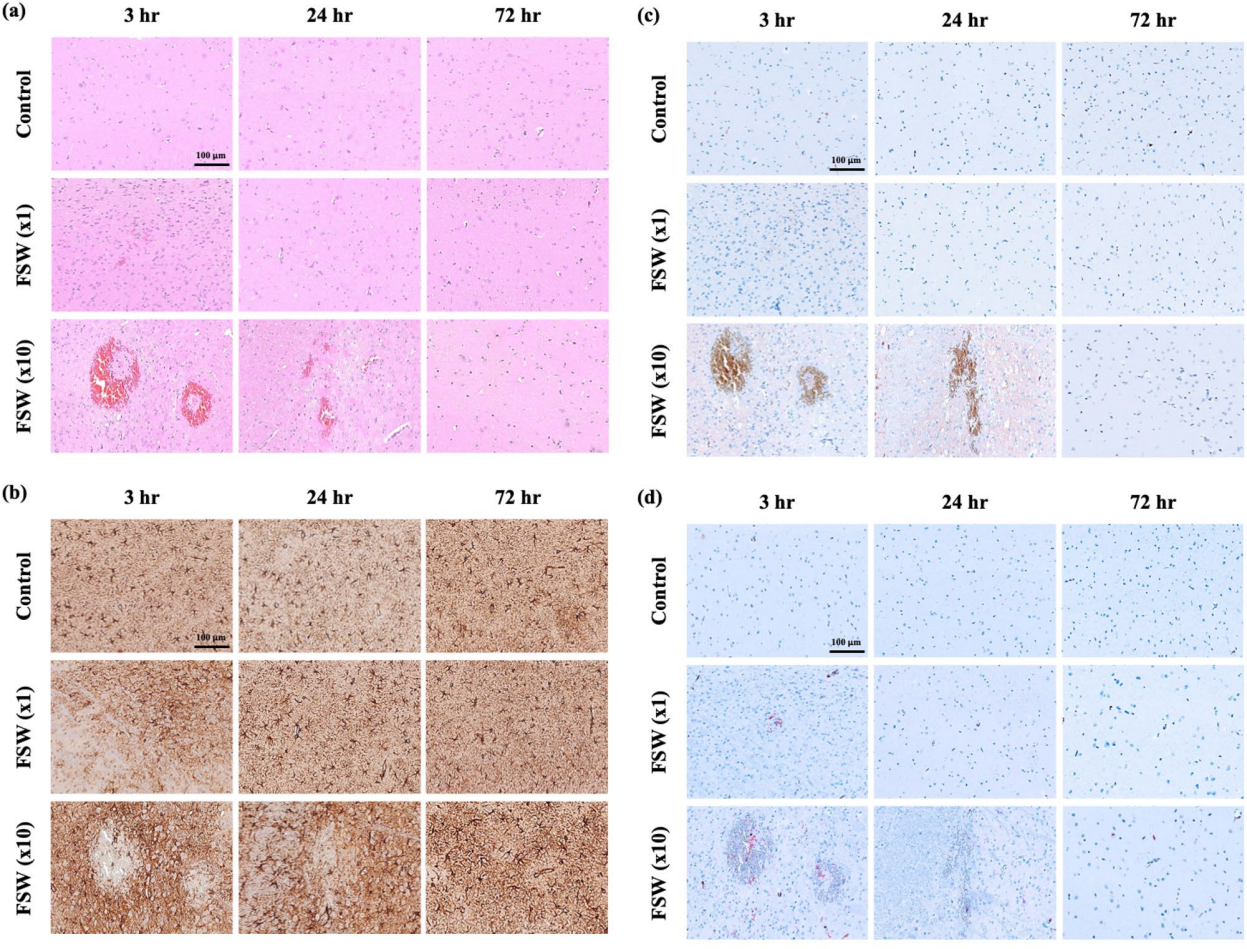

**Figure:**
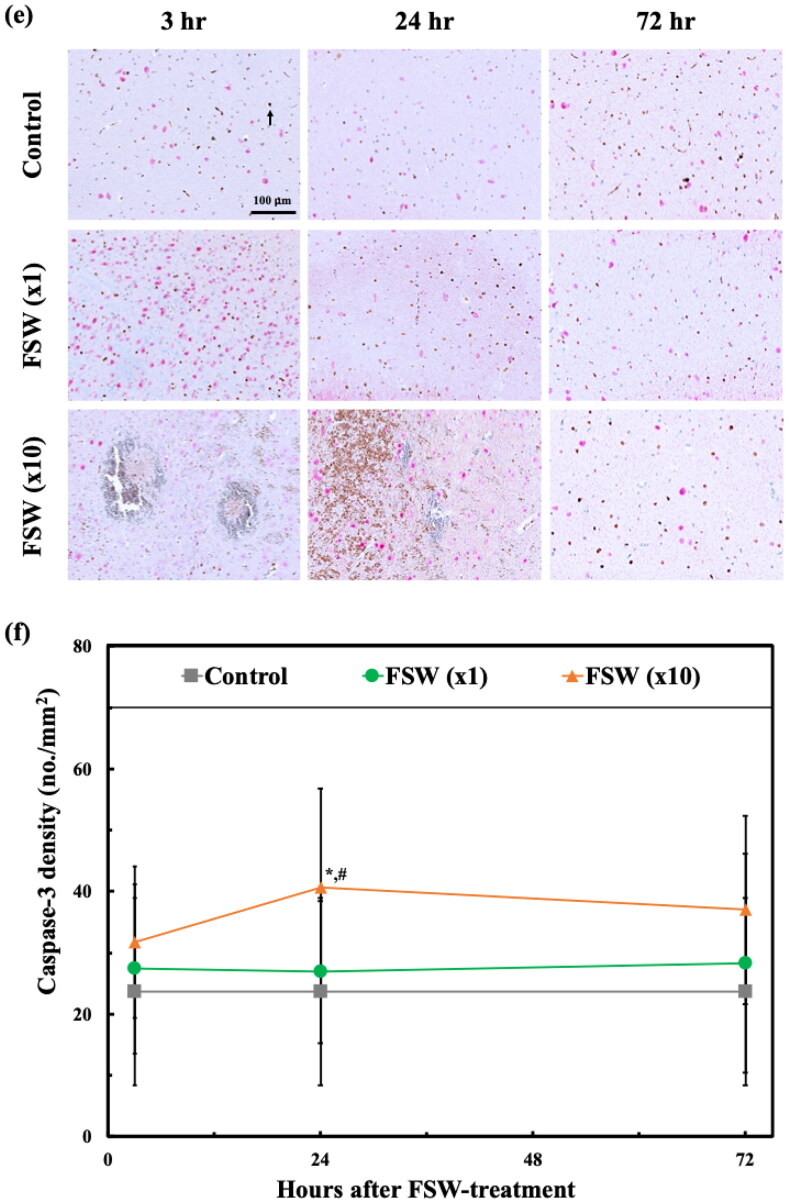

### 3.7. Inflammation, oxidative stress, and apoptosis related biomarkers

Behaviors, physiological evaluations, MRI, and DWI analysis were followed by brain tissue analysis and tracking. Overall, 24 hr after the FSW (×10) treatment we observed statistically significant differences in IL-1β, SOD, CAT, GSH, and MDA expression, but not T-AOC, as compared with the control and FSW (×1) treatment groups (Figure 7).

Figure 7.Comparisons of time responses of inflammation, oxidative stress and apoptosis-related biomarkers. (a) IL-1β expression; (b) SOD expression; (c) CAT expression; (d) GSH expression; (e) T-AOC expression; (f) MDA expression, in which, *, #*p* < 0.05 vs. control and vs. FSW (×1) groups, respectively, in one-way ANOVA with the Tukey post hoc test. (*n* = 5).

**Figure:**
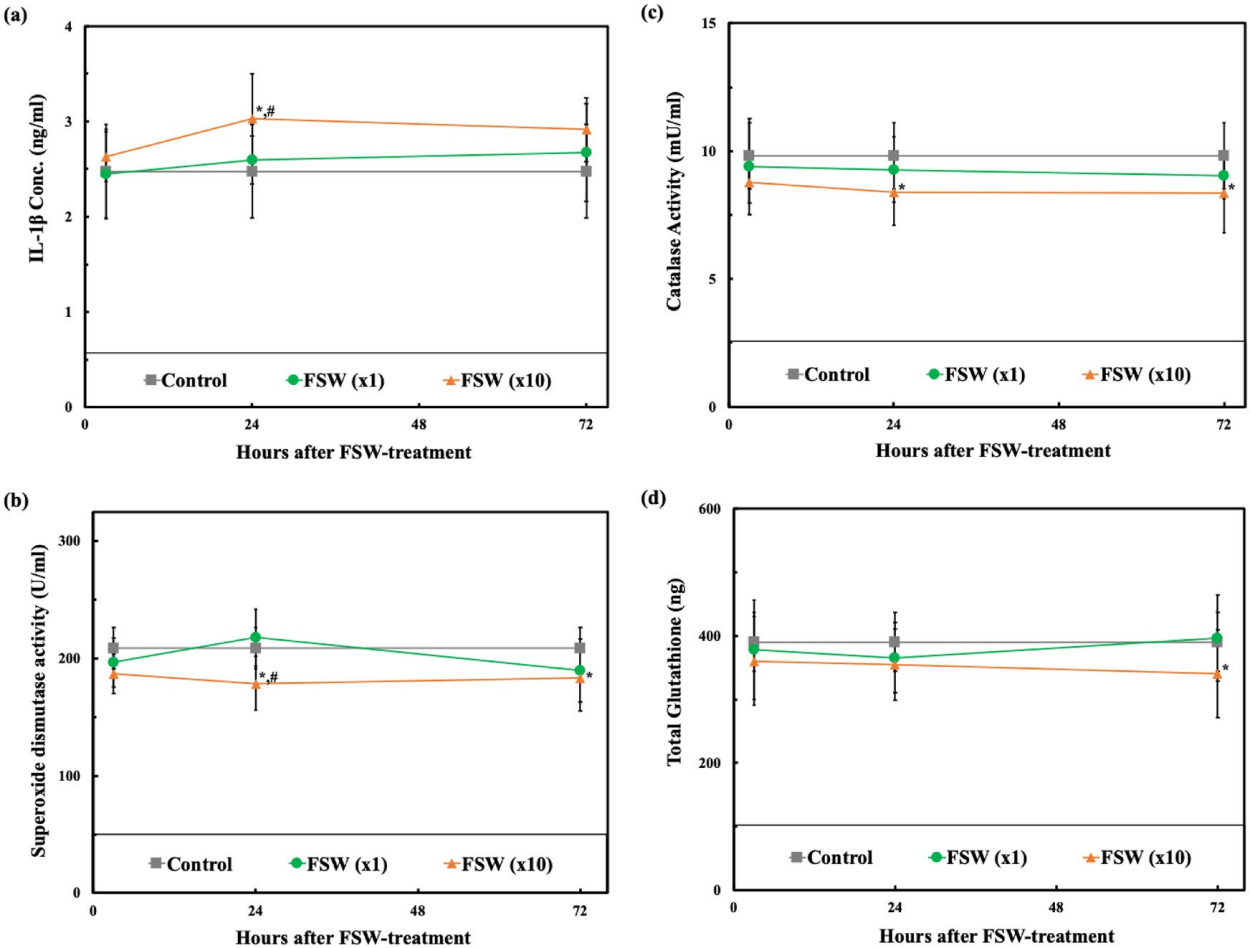

**Figure:**
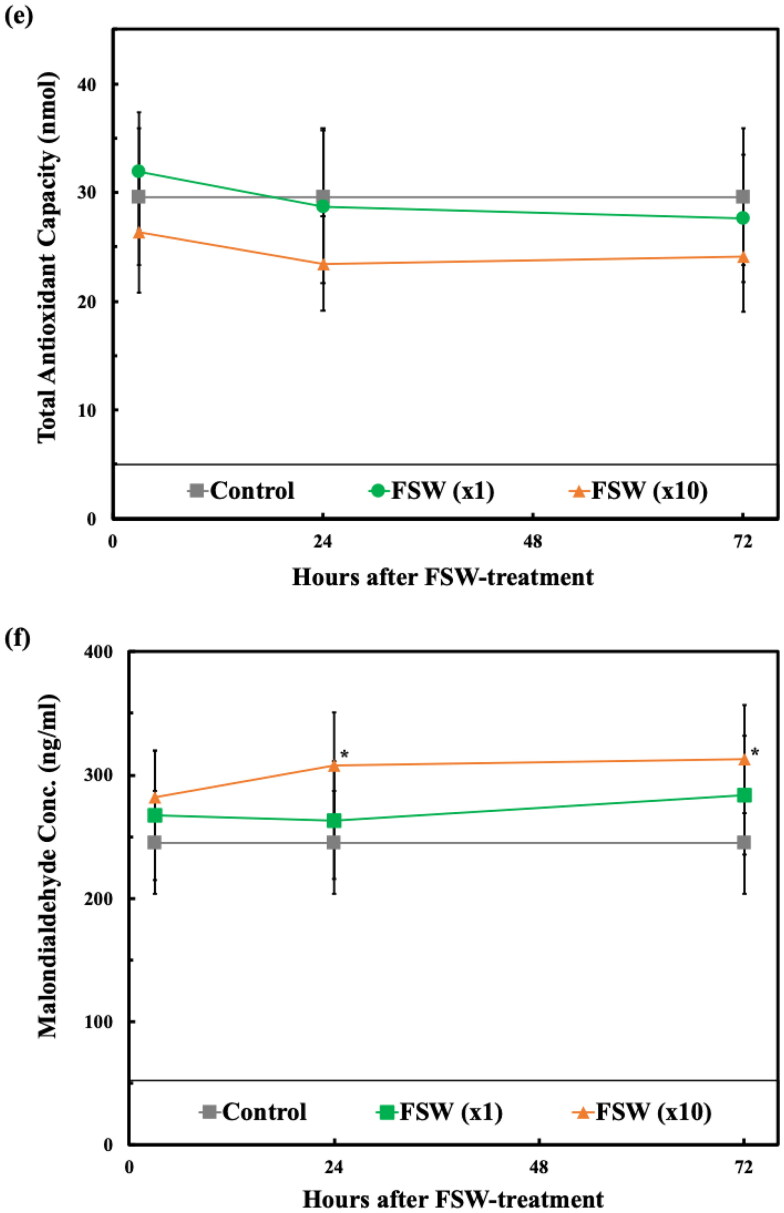

## 4. Discussion

The present study adjusted the targeting area 2.5 mm more superficially on the left lateral ventricle compared with previous studies (Paxinos & Watson, 1982; Kung et al., 2021). This modification lowers the microscopic hemorrhage rate from 60.0% to 20.0%, and the mortality rate from 20.0% to 0.0% even for 10 pulses of FSW. No microscopic hemorrhage or mortality was noted in single pulse FSW group while achieving similar BCSFB opening rate (100%). Although the exact reason for this difference of reduced hemorrhage rate is uncertain, we speculate that superficially focused FSW might lower the energy delivered to the underlying thalamus, a region found to have frequent hemorrhage after FSW using previous protocols (2.5 mm deeper).

The present study shows that the modified single pulse FSW technique, operating at an intensity effective for BCSFB opening, causes no observable behavioral changes, MRI-documented brain edema, or brain injury/inflammation histologically or immunologically. Even with 10 pulses of FSW, the resulting changes recovered within 72 hr, indicating the safety of the proposed method.

Our results showed no animal mortality, hypoactivity, ataxia, tremors, or moribundity occurred during the study period. In the behavior tests and physiological monitors, only 2/30 rats (6.67%) showed differences with others in terms of touch and click response at 3 hr after applying FSW (×10).

On the other hand, from H&E stains (Figure 7a), we could barely detect extravasations at 3 hr after FSW (×1) treatment, and only limited scale (around 100 micrometers) erythrocyte extravasations from 3 to 24 hr after FSW (×10) treatment. But these scale erythrocyte extravasations cannot be located on the MRI and DWI analysis (Figure 5). This may be caused by the limitations of slice thickness of MRI (Obenaus & Badaut, 2021).

With regard to microscopic injury, the proposed single-pulse FSW method showed no difference with the control group not only in terms of inflammation, oxidative stress, and apoptosis related biomarkers (Figure 6), but also on histopathologic and immunohistochemical sections (Figure 7) during the 3–72 hr of safety evaluation. However, the FSW (×10) group showed more apoptosis, inflammation, and oxidative stress on the immunohistochemical sections at the first 24 hr (Figure 7b–f), but no changes could be observed in the biomarkers and immunohistochemical sections at 72 hr, indicating a mild and reversible adverse effect even at 10 times the dosage of the proposed FSW-BCSFB opening method.

At present, drug delivery to CSF is effectively achieved by intrathecal delivery through lumbar puncture and intracerebroventricular (ICV) injection. However, these methods require invasive procedures (Rezai et al., 2020). Likewise, non-intrusive drug delivery to the CSF is accomplished by anti-transferrin receptor (TfR) antibody (OX-26 anti-rat TfR) (Zhang et al., 2021), by absorption into the lamina propria (Freyssin et al., 2018), by John Cunningham virus (JCV) (Steinhardt et al., 2020), and by nanoparticles with adoptive transfer ability of the BCSFB (Peviani et al., 2019). However, common disadvantages of these non-intrusive methods include immunogenic and inadequate delivery rates (Alexander et al., 2019). A safe alternative that can efficiently disrupt the BCSFB for drug delivery is crucially desired.

Few studies have examined the safety profiles of FSW-BBB or BCSFB opening. FUS shows early promise in clinical BBB opening and has been extensively evaluated for safety, and FUS-induced BBB opening can last for several hours at local and small regions (Carpentier et al., 2016). Meanwhile, the extravasation of albumin and red blood cells, and the generation of free radicals can be found in FUS treated regions (McDannold et al., 2005). In addition, FUS-induced cavitation of microbubbles still poses a risk of tissue injury and inflammatory response has been observed (e.g. infiltration, activation of CD4+ and CD8+ T cells, and cytotoxicity of Cytotoxic T lymphocytes), and brain hemorrhage (Aryal et al., 2014; Fan et al., 2014; van den Bijgaart et al., 2017). It has also been reported that thermal injury might result in brain regions receiving FUS (O’Reilly et al., 2010). The power required for FUS-BBB opening is around 25 J, which may increase focal temperature to around 7 °C, raising a potential risk for cranial nerve and tissue damage (Harnof et al., 2013; Palorini et al., 2015).

In contrast, FSW works at a lower frequency than HIPU and LIFU, producing less transcranial attenuation and better penetration (Kung et al., 2020). Because only a single, short-duration FSW pulse is required for BBB or BFCSB opening, there is negligible thermal effect on target area. There is only 0.6 °C maximal temperature rise at target area even after 3000 pulses of FSW stimulation (PRF 1 Hz, *P* + 80 MPa) (Wang et al., 2016),. Using a single FSW pulse, the total energy required for BBB opening is only 1/60,000 that produced by pulsed HIFU, and the requirement of microbubbles is significantly reduced to 1/5 of clinical dosage, and achieving a better successful opening rate, further reducing the risk of FSW-induced injury. Previous studies have indicated that single pulse FSW-BBB opening is associated with much lower free radical generation and reduced cellular apoptotic response compared to FUS-BBB opening, indicating that FSW is potentially a safer technique (Harada et al., 2013; Kung et al., 2020).

This study used the lowest possible commercial FSW intensity setting available. Considering the morphological domains between the BBB and BCSFB, it is possible that there is a lower intensity threshold for BCSFB opening (Solar et al., 2020), which would further reduce concerns of adverse effects such as hemorrhage and neuronal injury.

By fine-tuning the focal region, FSW-barrier opening can be controlled on the BBB or BCSFB, depending on the application purposes. Once the BCSFB has been opened by FSW, CSF drug concentrations in the brain and the spinal cord were markedly increased (Kung et al., 2021, 2022). Comparisons of different FSW-barrier openings are summarized in Table 3.

Based on the above, the modified single pulse FSW technique effectively opens BCSFB with minimal adverse effects, providing an effective and safe approach to deliver medications to the brain.

## 5. Conclusions

We have demonstrated the modified FSW-BCSFB opening by moving the target point 2.5 mm superficially is effective and safe. A single FSW pulse induced adequate BCSFB opening without causing cellular apoptosis, tissue damage or behavioral change. While 10 FSW pulses caused minor and transient behavior change and cellular apoptosis for the first few hours, the situation recovered after 72 hours. Therefore, the FSW-BCSFB opening technique presents a safe and effective alternative of CNS drug delivery, featuring FSW-triggered control widespread medication transport. Novel therapies for various CNS diseases could be developed based on this technique.

## Supplementary Material

Supplemental Material

## Data Availability

The datasets used and/or analyzed during the current study available from the corresponding author on reasonable request.
