# Supplementary material for: Blood-cerebrospinal fluid barrier opening by modified single pulse transcranial focused shockwave
Source: Drug Deliv. 2022 Dec 19;30(1):97–107. doi: 10.1080/10717544.2022.2157068 (PMC9769131; doi:10.1080/10717544.2022.2157068)
Supplement: Supplemental Material [file IDRD_A_2157068_SM9867.docx]

**Blood-cerebrospinal fluid barrier opening by modified single pulse transcranial focused shockwave**

Yi Kung^a^, Chueh-Hung Wu^b,c^, Meng Ting Lin^c^, Wei-Hao Liao^b^, Wen-Shiang Chen^b,d^, Ming-Yen Hsiao^b,*^

^a^Department of Biomechatronic Engineering, National Chiayi University, Chiayi city, Taiwan.

^b^Department of Physical Medicine and Rehabilitation, National Taiwan University Hospital & National Taiwan University College of Medicine, Taipei city, Taiwan.

^c^Department of Physical Medicine and Rehabilitation, National Taiwan University Hospital Hsin-Chu Branch, Hsinchu, Taiwan.

^d^Institute of Biomedical Engineering and Nanomedicine, National Health Research Institutes, Miaoli, Taiwan.

*Corresponding author

Ming-Yen Hsiao, E-mail: myferrant@gmail.com

**Supplementary material**

**Table**

**Table S1a. Summary of mortality and clinical observations**

|  |  | **Control** | | | | | | | | | **FSW-BCSFBO (1 time)** | | | | | | | | | **FSW-BCSFBO (10 times)** | | | | | | | | |
| --- | --- | --- | --- | --- | --- | --- | --- | --- | --- | --- | --- | --- | --- | --- | --- | --- | --- | --- | --- | --- | --- | --- | --- | --- | --- | --- | --- | --- |
| Item | Unit | n = 30 | | | n = 20 | | | n = 10 | | | n = 30 | | | n = 20 | | | n = 10 | | | n = 30 | | | n = 20 | | | n = 10 | | |
| Time | Hr | 3 | | | 24 | | | 72 | | | 3 | | | 24 | | | 72 | | | 3 | | | 24 | | | 72 | | |
| Mortality | rats | 0 | / | 30 | 0 | / | 20 | 0 | / | 10 | 0 | / | 30 | 0 | / | 20 | 0 | / | 10 | 0 | / | 30 | 0 | / | 20 | 0 | / | 10 |
| Hypoactivity | rats | 0 | / | 30 | 0 | / | 20 | 0 | / | 10 | 0 | / | 30 | 0 | / | 20 | 0 | / | 10 | 0 | / | 30 | 0 | / | 20 | 0 | / | 10 |
| Ataxia | rats | 0 | / | 30 | 0 | / | 20 | 0 | / | 10 | 0 | / | 30 | 0 | / | 20 | 0 | / | 10 | 0 | / | 30 | 0 | / | 20 | 0 | / | 10 |
| Tremors | rats | 0 | / | 30 | 0 | / | 20 | 0 | / | 10 | 0 | / | 30 | 0 | / | 20 | 0 | / | 10 | 0 | / | 30 | 0 | / | 20 | 0 | / | 10 |
| Swelling-area of forehead | rats | 0 | / | 30 | 0 | / | 20 | 0 | / | 10 | 0 | / | 30 | 0 | / | 20 | 0 | / | 10 | 0 | / | 30 | 0 | / | 20 | 0 | / | 10 |
| Urine stain | rats | 0 | / | 30 | 0 | / | 20 | 0 | / | 10 | 0 | / | 30 | 0 | / | 20 | 0 | / | 10 | 0 | / | 30 | 0 | / | 20 | 0 | / | 10 |
| Chromo-dacryorrhea | rats | 0 | / | 30 | 0 | / | 20 | 0 | / | 10 | 0 | / | 30 | 0 | / | 20 | 0 | / | 10 | 0 | / | 30 | 0 | / | 20 | 0 | / | 10 |
| Wounds-area of forehead | rats | 0 | / | 30 | 0 | / | 20 | 0 | / | 10 | 0 | / | 30 | 0 | / | 20 | 0 | / | 10 | 0 | / | 30 | 0 | / | 20 | 0 | / | 10 |
| Hair loss-forepaw | rats | 0 | / | 30 | 0 | / | 20 | 0 | / | 10 | 0 | / | 30 | 0 | / | 20 | 0 | / | 10 | 0 | / | 30 | 0 | / | 20 | 0 | / | 10 |

**Table S1b. Summary of descriptive endpoints during observation period**

|  |  | **Control** | | | | | | | | | **FSW-BCSFBO (1 time)** | | | | | | | | | **FSW-BCSFBO (10 times)** | | | | | | | | | |
| --- | --- | --- | --- | --- | --- | --- | --- | --- | --- | --- | --- | --- | --- | --- | --- | --- | --- | --- | --- | --- | --- | --- | --- | --- | --- | --- | --- | --- | --- |
| Item | Unit | n = 30 | | | n = 20 | | | n = 10 | | | n = 30 | | | n = 20 | | | n = 10 | | | n = 30 | | | n = 20 | | | n = 10 | | | |
| Time | Hr | 3 | | | 24 | | | 72 | | | 3 | | | 24 | | | 72 | | | 3 | | | 24 | | | 72 | | | |
| Posture: |  |  |  |  |  |  |  |  |  |  |  |  |  |  |  |  |  |  |  |  |  |  |  |  |  |  |  |  |  |
| Sitting | rats | 30 | / | 30 | 20 | / | 20 | 10 | / | 10 | 30 | / | 30 | 20 | / | 20 | 10 | / | 10 | 30 | / | 30 | 20 | / | 20 | 10 | / | 10 |  |
| Standing | rats | 30 | / | 30 | 20 | / | 20 | 10 | / | 10 | 30 | / | 30 | 20 | / | 20 | 10 | / | 10 | 30 | / | 30 | 20 | / | 20 | 10 | / | 10 |  |
| Alert | rats | 0 | / | 30 | 0 | / | 20 | 0 | / | 10 | 0 | / | 30 | 0 | / | 20 | 0 | / | 10 | 0 | / | 30 | 0 | / | 20 | 0 | / | 10 |  |
| Asleep | rats | 0 | / | 30 | 0 | / | 20 | 0 | / | 10 | 0 | / | 30 | 0 | / | 20 | 0 | / | 10 | 0 | / | 30 | 0 | / | 20 | 0 | / | 10 |  |
|  |  |  |  |  |  |  |  |  |  |  |  |  |  |  |  |  |  |  |  |  |  |  |  |  |  |  |  |  |  |
| Stereotype: |  |  |  |  |  |  |  |  |  |  |  |  |  |  |  |  |  |  |  |  |  |  |  |  |  |  |  |  |  |
| Tremor | rats | 0 | / | 30 | 0 | / | 20 | 0 | / | 10 | 0 | / | 30 | 0 | / | 20 | 0 | / | 10 | 0 | / | 30 | 0 | / | 20 | 0 | / | 10 |  |
|  |  |  |  |  |  |  |  |  |  |  |  |  |  |  |  |  |  |  |  |  |  |  |  |  |  |  |  |  |  |
| Bizarre behaviors: |  |  |  |  |  |  |  |  |  |  |  |  |  |  |  |  |  |  |  |  |  |  |  |  |  |  |  |  |  |
| Tremor | rats | 0 | / | 30 | 0 | / | 20 | 0 | / | 10 | 0 | / | 30 | 0 | / | 20 | 0 | / | 10 | 0 | / | 30 | 0 | / | 20 | 0 | / | 10 |  |

**Table S1c. Summary of categorical endpoints during observation period**

|  |  | **Control** | | | | | | | | | **FSW-BCSFBO (1 time)** | | | | | | | | | **FSW-BCSFBO (10 times)** | | | | | | | | | |
| --- | --- | --- | --- | --- | --- | --- | --- | --- | --- | --- | --- | --- | --- | --- | --- | --- | --- | --- | --- | --- | --- | --- | --- | --- | --- | --- | --- | --- | --- |
| Item | Unit | n = 30 | | | n = 20 | | | n = 10 | | | n = 30 | | | n = 20 | | | n = 10 | | | n = 30 | | | n = 20 | | | n = 10 | | |  |
| Time | Hr | 3 | | | 24 | | | 72 | | | 3 | | | 24 | | | 72 | | | 3 | | | 24 | | | 72 | | |  |
| Respiration | rats | 1.00 | ± | 0.00 | 1.00 | ± | 0.00 | 1.00 | ± | 0.00 | 1.00 | ± | 0.00 | 1.00 | ± | 0.00 | 1.00 | ± | 0.00 | 1.00 | ± | 0.00 | 1.00 | ± | 0.00 | 1.00 | ± | 0.00 |  |
| Clonic-involuntary | rats | 1.00 | ± | 0.00 | 1.00 | ± | 0.00 | 1.00 | ± | 0.00 | 1.00 | ± | 0.00 | 1.00 | ± | 0.00 | 1.00 | ± | 0.00 | 1.00 | ± | 0.00 | 1.00 | ± | 0.00 | 1.00 | ± | 0.00 |  |
| Tonic-involuntary | rats | 1.00 | ± | 0.00 | 1.00 | ± | 0.00 | 1.00 | ± | 0.00 | 1.00 | ± | 0.00 | 1.00 | ± | 0.00 | 1.00 | ± | 0.00 | 1.00 | ± | 0.00 | 1.00 | ± | 0.00 | 1.00 | ± | 0.00 |  |
| Vocali-zations | rats | 1.00 | ± | 0.00 | 1.00 | ± | 0.00 | 1.00 | ± | 0.00 | 1.00 | ± | 0.00 | 1.00 | ± | 0.00 | 1.00 | ± | 0.00 | 1.00 | ± | 0.00 | 1.00 | ± | 0.00 | 1.00 | ± | 0.00 |  |
| Piloerection | rats | 1.00 | ± | 0.00 | 1.00 | ± | 0.00 | 1.00 | ± | 0.00 | 1.00 | ± | 0.00 | 1.00 | ± | 0.00 | 1.00 | ± | 0.00 | 1.00 | ± | 0.00 | 1.00 | ± | 0.00 | 1.00 | ± | 0.00 |  |
| Palpebral closure | rats | 1.00 | ± | 0.00 | 1.00 | ± | 0.00 | 1.00 | ± | 0.00 | 1.00 | ± | 0.00 | 1.00 | ± | 0.00 | 1.00 | ± | 0.00 | 1.00 | ± | 0.00 | 1.00 | ± | 0.00 | 1.00 | ± | 0.00 |  |
| Ease to remove | rats | 3.00 | ± | 0.00 | 3.00 | ± | 0.00 | 3.00 | ± | 0.00 | 3.00 | ± | 0.00 | 3.00 | ± | 0.00 | 3.00 | ± | 0.00 | 3.00 | ± | 0.00 | 3.00 | ± | 0.00 | 3.00 | ± | 0.00 |  |
| Ease to handle | rats | 3.00 | ± | 0.00 | 3.00 | ± | 0.00 | 3.00 | ± | 0.00 | 3.00 | ± | 0.00 | 3.00 | ± | 0.00 | 3.00 | ± | 0.00 | 3.00 | ± | 0.00 | 3.00 | ± | 0.00 | 3.00 | ± | 0.00 |  |
| Chromodacryorrhea | rats | 1.00 | ± | 0.00 | 1.00 | ± | 0.00 | 1.00 | ± | 0.00 | 1.00 | ± | 0.00 | 1.00 | ± | 0.00 | 1.00 | ± | 0.00 | 1.00 | ± | 0.00 | 1.00 | ± | 0.00 | 1.00 | ± | 0.00 |  |
| Lacrimation | rats | 1.00 | ± | 0.00 | 1.00 | ± | 0.00 | 1.00 | ± | 0.00 | 1.00 | ± | 0.00 | 1.00 | ± | 0.00 | 1.00 | ± | 0.00 | 1.00 | ± | 0.00 | 1.00 | ± | 0.00 | 1.00 | ± | 0.00 |  |
| Salivation | rats | 1.00 | ± | 0.00 | 1.00 | ± | 0.00 | 1.00 | ± | 0.00 | 1.00 | ± | 0.00 | 1.00 | ± | 0.00 | 1.00 | ± | 0.00 | 1.00 | ± | 0.00 | 1.00 | ± | 0.00 | 1.00 | ± | 0.00 |  |
| Fur coat | rats | 1.00 | ± | 0.00 | 1.00 | ± | 0.00 | 1.00 | ± | 0.00 | 1.00 | ± | 0.00 | 1.00 | ± | 0.00 | 1.00 | ± | 0.00 | 1.00 | ± | 0.00 | 1.00 | ± | 0.00 | 1.00 | ± | 0.00 |  |
| Visual approach response | rats | 1.00 | ± | 0.00 | 1.00 | ± | 0.00 | 1.00 | ± | 0.00 | 1.00 | ± | 0.00 | 1.00 | ± | 0.00 | 1.00 | ± | 0.00 | 1.00 | ± | 0.00 | 1.00 | ± | 0.00 | 1.00 | ± | 0.00 |  |
| Touch response | rats | 1.00 | ± | 0.00 | 1.00 | ± | 0.00 | 1.00 | ± | 0.00 | 1.00 | ± | 0.00 | 1.00 | ± | 0.00 | 1.00 | ± | 0.00 | 1.07 | ± | 0.25 | 1.00 | ± | 0.00 | 1.00 | ± | 0.00 |  |
| Click response | rats | 1.00 | ± | 0.00 | 1.00 | ± | 0.00 | 1.00 | ± | 0.00 | 1.00 | ± | 0.00 | 1.00 | ± | 0.00 | 1.00 | ± | 0.00 | 1.07 | ± | 0.25 | 1.00 | ± | 0.00 | 1.00 | ± | 0.00 |  |
| Eyelid reflex | rats | 1.00 | ± | 0.00 | 1.00 | ± | 0.00 | 1.00 | ± | 0.00 | 1.00 | ± | 0.00 | 1.00 | ± | 0.00 | 1.00 | ± | 0.00 | 1.00 | ± | 0.00 | 1.00 | ± | 0.00 | 1.00 | ± | 0.00 |  |
| Pinna reflex | rats | 1.00 | ± | 0.00 | 1.00 | ± | 0.00 | 1.00 | ± | 0.00 | 1.00 | ± | 0.00 | 1.00 | ± | 0.00 | 1.00 | ± | 0.00 | 1.00 | ± | 0.00 | 1.00 | ± | 0.00 | 1.00 | ± | 0.00 |  |
| Tail pinch response | rats | 1.00 | ± | 0.00 | 1.00 | ± | 0.00 | 1.00 | ± | 0.00 | 1.00 | ± | 0.00 | 1.00 | ± | 0.00 | 1.00 | ± | 0.00 | 1.00 | ± | 0.00 | 1.00 | ± | 0.00 | 1.00 | ± | 0.00 |  |
| Pupil reflex | rats | 1.00 | ± | 0.00 | 1.00 | ± | 0.00 | 1.00 | ± | 0.00 | 1.00 | ± | 0.00 | 1.00 | ± | 0.00 | 1.00 | ± | 0.00 | 1.00 | ± | 0.00 | 1.00 | ± | 0.00 | 1.00 | ± | 0.00 |  |
| Proprioception | rats | 1.00 | ± | 0.00 | 1.00 | ± | 0.00 | 1.00 | ± | 0.00 | 1.00 | ± | 0.00 | 1.00 | ± | 0.00 | 1.00 | ± | 0.00 | 1.00 | ± | 0.00 | 1.00 | ± | 0.00 | 1.00 | ± | 0.00 |  |
| Air righting reflex | rats | 1.00 | ± | 0.00 | 1.00 | ± | 0.00 | 1.00 | ± | 0.00 | 1.00 | ± | 0.00 | 1.00 | ± | 0.00 | 1.00 | ± | 0.00 | 1.00 | ± | 0.00 | 1.00 | ± | 0.00 | 1.00 | ± | 0.00 |  |
| Abdominal tone | rats | 3.00 | ± | 0.00 | 3.00 | ± | 0.00 | 3.00 | ± | 0.00 | 3.00 | ± | 0.00 | 3.00 | ± | 0.00 | 3.00 | ± | 0.00 | 3.00 | ± | 0.00 | 3.00 | ± | 0.00 | 3.00 | ± | 0.00 |  |
| Limb tone | rats | 3.00 | ± | 0.00 | 3.00 | ± | 0.00 | 3.00 | ± | 0.00 | 3.00 | ± | 0.00 | 3.00 | ± | 0.00 | 3.00 | ± | 0.00 | 3.00 | ± | 0.00 | 3.00 | ± | 0.00 | 3.00 | ± | 0.00 |  |
| Grip strength | rats | 3.00 | ± | 0.00 | 3.00 | ± | 0.00 | 3.00 | ± | 0.00 | 3.00 | ± | 0.00 | 3.00 | ± | 0.00 | 3.00 | ± | 0.00 | 3.00 | ± | 0.00 | 3.00 | ± | 0.00 | 3.00 | ± | 0.00 |  |
| Gait | rats | 1.00 | ± | 0.00 | 1.00 | ± | 0.00 | 1.00 | ± | 0.00 | 1.00 | ± | 0.00 | 1.00 | ± | 0.00 | 1.00 | ± | 0.00 | 1.00 | ± | 0.00 | 1.00 | ± | 0.00 | 1.00 | ± | 0.00 |  |
| Mobility | rats | 1.00 | ± | 0.00 | 1.00 | ± | 0.00 | 1.00 | ± | 0.00 | 1.00 | ± | 0.00 | 1.00 | ± | 0.00 | 1.00 | ± | 0.00 | 1.00 | ± | 0.00 | 1.00 | ± | 0.00 | 1.00 | ± | 0.00 |  |
| Arousal | rats | 1.00 | ± | 0.00 | 1.00 | ± | 0.00 | 1.00 | ± | 0.00 | 1.00 | ± | 0.00 | 1.00 | ± | 0.00 | 1.00 | ± | 0.00 | 1.00 | ± | 0.00 | 1.00 | ± | 0.00 | 1.00 | ± | 0.00 |  |
| Exophthalmus | rats | 1.00 | ± | 0.00 | 1.00 | ± | 0.00 | 1.00 | ± | 0.00 | 1.00 | ± | 0.00 | 1.00 | ± | 0.00 | 1.00 | ± | 0.00 | 1.00 | ± | 0.00 | 1.00 | ± | 0.00 | 1.00 | ± | 0.00 |  |

**Table S2a. Summary of body measurements**

|  |  | **Control** | | | | | | | | | **FSW-BCSFBO (1 time)** | | | | | | | | | **FSW-BCSFBO (10 times)** | | | | | | | | | |
| --- | --- | --- | --- | --- | --- | --- | --- | --- | --- | --- | --- | --- | --- | --- | --- | --- | --- | --- | --- | --- | --- | --- | --- | --- | --- | --- | --- | --- | --- |
| Time | Hr | 3 | | | 24 | | | 72 | | | 3 | | | 24 | | | 72 | | | 3 | | | 24 | | | 72 | | |  |
| Item | Unit | n = 30 | | | n = 20 | | | n = 10 | | | n = 30 | | | n = 20 | | | n = 10 | | | n = 30 | | | n = 20 | | | n = 10 | | |  |
| Consume: |  |  |  |  |  |  |  |  |  |  |  |  |  |  |  |  |  |  |  |  |  |  |  |  |  |  |  |  |  |
| Water | mL | 45.09 | ± | 7.11 | 50.18 | ± | 6.14 | 48.45 | ± | 6.59 | 49.18 | ± | 7.25 | 48.45 | ± | 7.80 | 47.55 | ± | 5.14 | 44.73 | ± | 7.80 | 46.82 | ± | 8.49 | 49.09 | ± | 6.28 |  |
| Fodder | g | 21.36 | ± | 6.34 | 26.64 | ± | 5.85 | 27.91 | ± | 9.14 | 25.57 | ± | 8.26 | 24.18 | ± | 8.36 | 26.55 | ± | 6.42 | 27.18 | ± | 5.80 | 25.36 | ± | 6.90 | 28.64 | ± | 8.87 |  |
|  |  |  |  |  |  |  |  |  |  |  |  |  |  |  |  |  |  |  |  |  |  |  |  |  |  |  |  |  |  |
| Body: |  |  |  |  |  |  |  |  |  |  |  |  |  |  |  |  |  |  |  |  |  |  |  |  |  |  |  |  |  |
| Temperature | ^o^C | 37.27 | ± | 1.19 | 36.91 | ± | 1.38 | 36.36 | ± | 1.36 | 36.18 | ± | 1.47 | 36.64 | ± | 1.80 | 36.27 | ± | 1.85 | 37.45 | ± | 0.93 | 36.73 | ± | 1.49 | 36.18 | ± | 1.47 |  |
| Weight | g | 245.56 | ± | 16.74 | 258.00 | ± | 22.78 | 259.45 | ± | 23.65 | 254.80 | ± | 12.68 | 260.36 | ± | 19.94 | 258.45 | ± | 22.33 | 250.45 | ± | 21.90 | 260.91 | ± | 19.12 | 262.45 | ± | 18.64 |  |
|  |  |  |  |  |  |  |  |  |  |  |  |  |  |  |  |  |  |  |  |  |  |  |  |  |  |  |  |  |  |
| Brain: |  | n = 10 | | | n = 10 | | | n = 10 | | | n = 10 | | | n = 10 | | | n = 10 | | | n = 10 | | | n = 10 | | | n = 10 | | |  |
| Weight | g | 2.12 | ± | 0.09 | 2.06 | ± | 0.09 | 2.13 | ± | 0.08 | 2.10 | ± | 0.09 | 2.12 | ± | 0.07 | 2.14 | ± | 0.08 | 2.12 | ± | 0.09 | 2.08 | ± | 0.08 | 2.11 | ± | 0.08 |  |

**Table S2b. Summary of serum biochemistry**

|  |  | **Control** | | | | | | | | | **FSW-BCSFBO (1 time)** | | | | | | | | | **FSW-BCSFBO (10 times)** | | | | | | | | |
| --- | --- | --- | --- | --- | --- | --- | --- | --- | --- | --- | --- | --- | --- | --- | --- | --- | --- | --- | --- | --- | --- | --- | --- | --- | --- | --- | --- | --- |
| Item | Unit | n = 10 | | | n = 10 | | | n = 10 | | | n = 10 | | | n = 10 | | | n = 10 | | | n = 10 | | | n = 10 | | | n = 10 | | |
| Time | Hr | 3 | | | 24 | | | 72 | | | 3 | | | 24 | | | 72 | | | 3 | | | 24 | | | 72 | | |
| ALB | g/L | 43.52 | ± | 2.78 | 41.53 | ± | 2.66 | 45.50 | ± | 0.62 | 43.03 | ± | 2.58 | 43.03 | ± | 2.67 | 42.20 | ± | 3.39 | 42.08 | ± | 2.00 | 41.52 | ± | 1.27 | 41.72 | ± | 1.37 |
| ALT | U/L | 42.00 | ± | 3.74 | 41.33 | ± | 3.51 | 42.67 | ± | 4.62 | 47.33 | ± | 4.39 | 47.33 | ± | 4.04 | 52.50 | ± | 3.54 | 43.20 | ± | 12.79 | 38.64 | ± | 2.78 | 39.30 | ± | 2.73 |
| AST | U/L | 91.78 | ± | 10.69 | 97.10 | ± | 3.70 | 86.47 | ± | 13.67 | 97.67 | ± | 8.42 | 97.67 | ± | 7.97 | 108.60 | ± | 3.68 | 86.78 | ± | 16.71 | 80.98 | ± | 5.17 | 79.77 | ± | 5.09 |
| BIL-T | mg/dL | 0.03 | ± | 0.02 | 0.02 | ± | 0.02 | 0.03 | ± | 0.01 | 0.05 | ± | 0.04 | 0.05 | ± | 0.04 | 0.01 | ± | 0.02 | 0.00 | ± | 0.01 | 0.01 | ± | 0.01 | 0.00 | ± | 0.00 |
| BUN | mg/dL | 13.51 | ± | 1.59 | 14.42 | ± | 0.99 | 12.60 | ± | 1.69 | 10.45 | ± | 1.79 | 10.45 | ± | 1.69 | 9.43 | ± | 2.41 | 13.58 | ± | 2.07 | 13.02 | ± | 1.38 | 12.57 | ± | 1.08 |
| T-Cho | mg/dL | 57.92 | ± | 5.53 | 56.77 | ± | 3.10 | 59.07 | ± | 7.92 | 51.30 | ± | 12.00 | 51.30 | ± | 8.65 | 69.55 | ± | 5.16 | 49.00 | ± | 7.57 | 49.56 | ± | 7.41 | 50.20 | ± | 8.40 |
| Crea | mg/dL | 0.28 | ± | 0.04 | 0.25 | ± | 0.03 | 0.31 | ± | 0.02 | 0.28 | ± | 0.05 | 0.28 | ± | 0.04 | 0.22 | ± | 0.06 | 0.24 | ± | 0.05 | 0.25 | ± | 0.05 | 0.24 | ± | 0.05 |
| GLU | mg/dL | 418.60 | ± | 113.21 | 341.75 | ± | 111.32 | 495.44 | ± | 43.94 | 399.15 | ± | 82.92 | 399.15 | ± | 81.24 | 324.10 | ± | 86.85 | 264.52 | ± | 25.05 | 271.17 | ± | 17.20 | 271.75 | ± | 19.81 |
| HDL | mg/dL | 43.42 | ± | 7.35 | 40.57 | ± | 2.85 | 46.26 | ± | 10.13 | 37.83 | ± | 7.64 | 37.83 | ± | 5.15 | 49.90 | ± | 2.32 | 34.80 | ± | 6.02 | 34.62 | ± | 6.00 | 35.07 | ± | 6.83 |
| LDL | mg/dL | 8.45 | ± | 1.20 | 8.99 | ± | 0.72 | 7.91 | ± | 1.49 | 12.18 | ± | 3.89 | 12.18 | ± | 4.69 | 14.61 | ± | 3.08 | 9.99 | ± | 2.53 | 10.73 | ± | 1.50 | 11.20 | ± | 1.25 |
| IP | mg/dL | 12.12 | ± | 0.79 | 11.79 | ± | 0.48 | 12.44 | ± | 1.01 | 11.45 | ± | 1.49 | 11.45 | ± | 1.84 | 11.61 | ± | 1.44 | 9.84 | ± | 1.20 | 9.97 | ± | 1.15 | 10.13 | ± | 1.26 |
| T-Rro | g/dL | 5.65 | ± | 0.24 | 5.50 | ± | 0.26 | 5.80 | ± | 0.10 | 5.47 | ± | 0.23 | 5.47 | ± | 0.25 | 5.50 | ± | 0.28 | 5.24 | ± | 0.25 | 5.17 | ± | 0.16 | 5.19 | ± | 0.17 |
| TG | mg/dL | 113.86 | ± | 53.23 | 133.75 | ± | 19.00 | 93.97 | ± | 74.40 | 52.02 | ± | 20.83 | 52.02 | ± | 14.74 | 75.91 | ± | 24.83 | 69.98 | ± | 15.23 | 71.76 | ± | 14.43 | 70.52 | ± | 16.35 |
| Na | mM/L | 147.17 | ± | 6.74 | 149.40 | ± | 4.39 | 150.43 | ± | 2.35 | 143.92 | ± | 7.47 | 143.92 | ± | 6.54 | 144.90 | ± | 7.11 | 140.20 | ± | 2.49 | 139.84 | ± | 2.29 | 139.30 | ± | 2.24 |
| K | mM/L | 5.43 | ± | 0.37 | 5.44 | ± | 0.42 | 5.35 | ± | 0.33 | 5.85 | ± | 1.07 | 5.85 | ± | 0.66 | 5.86 | ± | 0.77 | 4.98 | ± | 0.25 | 5.02 | ± | 0.23 | 5.05 | ± | 0.25 |
| Cl | mM/L | 119.33 | ± | 5.09 | 119.00 | ± | 5.61 | 119.07 | ± | 5.61 | 109.72 | ± | 3.29 | 109.72 | ± | 3.27 | 108.90 | ± | 3.13 | 103.20 | ± | 1.92 | 103.04 | ± | 1.87 | 102.55 | ± | 1.75 |

**Table S2c. Summary of hematology**

|  |  | **Control** | | | | | | | | | **FSW-BCSFBO (1 time)** | | | | | | | | | **FSW-BCSFBO (10 times)** | | | | | | | | |
| --- | --- | --- | --- | --- | --- | --- | --- | --- | --- | --- | --- | --- | --- | --- | --- | --- | --- | --- | --- | --- | --- | --- | --- | --- | --- | --- | --- | --- |
| Item | Unit | n = 10 | | | n = 10 | | | n = 10 | | | n = 10 | | | n = 10 | | | n = 10 | | | n = 10 | | | n = 10 | | | n = 10 | | |
| Time | Hr | 3 | | | 24 | | | 72 | | | 3 | | | 24 | | | 72 | | | 3 | | | 24 | | | 72 | | |
| RBC | M/uL | 6.11 | ± | 0.31 | 5.88 | ± | 0.23 | 6.27 | ± | 0.28 | 6.76 | ± | 0.29 | 6.75 | ± | 0.12 | 6.77 | ± | 0.40 | 6.50 | ± | 0.5 | 6.22 | ± | 0.5 | 6.76 | ± | 0.5 |
| HGB | g/dL | 13.10 | ± | 0.72 | 12.50 | ± | 0.57 | 13.50 | ± | 0.52 | 14.06 | ± | 0.83 | 14.05 | ± | 0.21 | 14.07 | ± | 1.17 | 13.67 | ± | 1.0 | 14.64 | ± | 1.0 | 14.81 | ± | 1.0 |
| HCT | % | 42.06 | ± | 3.24 | 38.90 | ± | 1.98 | 44.17 | ± | 1.55 | 46.26 | ± | 3.44 | 46.40 | ± | 1.27 | 46.17 | ± | 4.77 | 46.55 | ± | 3.6 | 46.26 | ± | 3.5 | 46.89 | ± | 3.5 |
| MCV | fL | 68.79 | ± | 2.94 | 66.20 | ± | 0.74 | 70.52 | ± | 2.41 | 68.34 | ± | 2.35 | 68.79 | ± | 0.66 | 68.04 | ± | 3.25 | 64.60 | ± | 2.2 | 64.02 | ± | 1.4 | 64.40 | ± | 1.2 |
| MCH | pg | 21.45 | ± | 0.69 | 21.27 | ± | 0.12 | 21.56 | ± | 0.95 | 20.78 | ± | 0.37 | 20.83 | ± | 0.06 | 20.75 | ± | 0.52 | 20.37 | ± | 0.3 | 20.28 | ± | 0.2 | 20.35 | ± | 0.1 |
| MCHC | g/dL | 31.19 | ± | 0.89 | 32.14 | ± | 0.18 | 30.56 | ± | 0.31 | 30.43 | ± | 0.69 | 30.29 | ± | 0.37 | 30.52 | ± | 0.92 | 31.52 | ± | 0.7 | 31.67 | ± | 0.6 | 31.60 | ± | 0.6 |
| RET | K/uL | 502.12 | ± | 46.61 | 485.65 | ± | 52.96 | 513.10 | ± | 49.91 | 498.70 | ± | 87.26 | 421.00 | ± | 56.57 | 550.50 | ± | 59.73 | 426.53 | ± | 91.3 | 416.67 | ± | 86.7 | 422.83 | ± | 95.5 |
| RET | % | 8.21 | ± | 0.53 | 8.25 | ± | 0.57 | 8.18 | ± | 0.62 | 7.37 | ± | 1.23 | 6.24 | ± | 0.73 | 8.13 | ± | 0.77 | 5.97 | ± | 1.5 | 5.82 | ± | 1.5 | 5.87 | ± | 1.6 |
| PLT | K/uL | 1039.40 | ± | 127.77 | 955.50 | ± | 157.68 | 1095.33 | ± | 92.12 | 1155.40 | ± | 133.18 | 1089.50 | ± | 127.99 | 1199.33 | ± | 141.58 | 1191.67 | ± | 157.5 | 1189.94 | ± | 167.4 | 1180.13 | ± | 185.2 |
| WBC | K/uL | 4.48 | ± | 1.07 | 3.77 | ± | 1.65 | 4.95 | ± | 0.27 | 5.40 | ± | 1.10 | 5.97 | ± | 0.62 | 6.01 | ± | 1.49 | 4.62 | ± | 0.9 | 4.30 | ± | 0.9 | 4.81 | ± | 0.8 |
| NEUT | K/uL | 0.68 | ± | 0.30 | 0.77 | ± | 0.37 | 0.62 | ± | 0.31 | 0.85 | ± | 0.20 | 0.81 | ± | 0.13 | 0.87 | ± | 0.25 | 0.47 | ± | 0.1 | 0.48 | ± | 0.1 | 0.49 | ± | 0.1 |
| LYMPH | K/uL | 3.27 | ± | 0.87 | 2.59 | ± | 1.05 | 3.73 | ± | 0.45 | 4.35 | ± | 0.48 | 4.40 | ± | 0.12 | 4.32 | ± | 0.68 | 3.30 | ± | 0.9 | 3.29 | ± | 0.9 | 3.06 | ± | 0.8 |
| MONO | K/uL | 0.43 | ± | 0.22 | 0.25 | ± | 0.13 | 0.56 | ± | 0.17 | 0.76 | ± | 0.53 | 0.74 | ± | 0.36 | 0.78 | ± | 0.71 | 0.23 | ± | 0.1 | 0.23 | ± | 0.1 | 0.24 | ± | 0.1 |
| EO | K/uL | 0.08 | ± | 0.09 | 0.15 | ± | 0.11 | 0.03 | ± | 0.01 | 0.02 | ± | 0.01 | 0.03 | ± | 0.01 | 0.02 | ± | 0.00 | 0.01 | ± | 0.0 | 0.01 | ± | 0.0 | 0.01 | ± | 0.0 |
| BASO | K/uL | 0.01 | ± | 0.01 | 0.02 | ± | 0.01 | 0.01 | ± | 0.01 | 0.01 | ± | 0.01 | 0.01 | ± | 0.01 | 0.02 | ± | 0.01 | 0.04 | ± | 0.1 | 0.05 | ± | 0.1 | 0.02 | ± | 0.0 |
| NEUT | % | 15.70 | ± | 6.26 | 20.15 | ± | 0.92 | 12.74 | ± | 6.71 | 14.17 | ± | 2.96 | 13.47 | ± | 0.89 | 14.63 | ± | 4.05 | 12.38 | ± | 5.3 | 12.98 | ± | 5.0 | 13.86 | ± | 5.1 |
| LYMPH | % | 72.78 | ± | 5.01 | 69.28 | ± | 2.65 | 75.12 | ± | 5.12 | 73.34 | ± | 6.41 | 73.91 | ± | 5.69 | 72.95 | ± | 8.08 | 81.32 | ± | 5.4 | 80.69 | ± | 5.1 | 79.48 | ± | 4.6 |
| MONO | % | 9.29 | ± | 3.70 | 6.33 | ± | 0.79 | 11.27 | ± | 3.52 | 11.86 | ± | 6.04 | 12.06 | ± | 4.78 | 11.73 | ± | 7.84 | 5.75 | ± | 1.2 | 5.74 | ± | 1.2 | 6.07 | ± | 0.9 |
| EO | % | 1.88 | ± | 1.79 | 3.67 | ± | 1.39 | 0.68 | ± | 0.14 | 0.40 | ± | 0.13 | 0.49 | ± | 0.19 | 0.35 | ± | 0.08 | 0.28 | ± | 0.1 | 0.28 | ± | 0.1 | 0.30 | ± | 0.1 |
| BASO | % | 0.31 | ± | 0.29 | 0.49 | ± | 0.40 | 0.20 | ± | 0.19 | 0.22 | ± | 0.21 | 0.09 | ± | 0.13 | 0.30 | ± | 0.24 | 0.27 | ± | 0.2 | 0.31 | ± | 0.1 | 0.29 | ± | 0.1 |
